# Understanding long-acting injectables in the context of treatment planning for schizophrenia: crafting a strategy

**DOI:** 10.1017/S1092852925100680

**Published:** 2025-12-19

**Authors:** Leslie Citrome, Desiree Matthews

**Affiliations:** 1 Department of Psychiatry and Behavioral Sciences, New York Medical College, USA; 2 Different MHP, PC, Charlotte, USA

**Keywords:** Adherence, long-acting injectable antipsychotic, schizophrenia, schizoaffective disorder, bipolar disorder, treatment planning

## Abstract

Long-acting injectable antipsychotics (LAIs) can lead to improved outcomes for people with schizophrenia, schizoaffective disorder, and bipolar disorder, as they guarantee medication delivery during the injection interval. Contemporary guidance on the use of LAIs includes considering not only patients with poor or uncertain adherence but also patients who would prefer monthly administration (or longer) of their maintenance medication, including those in their first episode of illness. This narrative review discusses the incorporation of LAIs in treatment planning across different settings: acute inpatient units, community mental health outpatient clinics, and jails. Implementing this treatment modality requires the recognition of eligible patients, providing information to patients and their families about the benefits and drawbacks of LAIs, and educating all members of the treatment team.

## Introduction

Antipsychotics serve as the foundational treatment for people with schizophrenia and schizoaffective disorder, as well as for many people living with bipolar disorder. As with almost all chronic medical conditions, fidelity to a daily medication regimen is challenging. This includes other disorders such as hypertension, diabetes, and asthma, as well as major depressive disorder, anxiety disorder, and attention-deficit/hyperactivity disorder.^1^ Lack of adequate adherence can occur quickly after hospital discharge; in a sample of 68 patients with schizophrenia, partial adherence was found in 25% of patients with schizophrenia within 2 weeks of their hospital discharge, increasing to 50% at 1 year and 75% at 2 years.^2^ Gaps in therapy can increase the rate of rehospitalization; in one study, patients who did not have gaps in medication therapy had a rate of rehospitalization of about 5% over a 1-year period, but patients who had a 1- to 10-day maximum gap had almost twice the odds of hospitalization.^3^ Other deleterious outcomes linked to poor adherence include higher rates of emergency psychiatric care, being arrested, being a victim of a crime, and substance misuse.^4^ Similar concerns apply to other severe psychiatric conditions, such as bipolar I disorder and schizoaffective disorder.^5^^–^^9^ Treatment planning with the aim of reducing relapse or recurrence can include consideration of long-acting injectable antipsychotics (LAIs), essentially guaranteeing medication delivery within the dosing interval. This narrative review describes the use of LAIs in different settings within the context of treatment planning: acute inpatient, community mental health outpatient clinics, and jails.

## Risk factors for poor adherence to antipsychotic medications influence treatment planning

Creating a personalized treatment plan requires a systematic assessment of an individual’s array of risk factors for poor adherence.^6^^,^^10^^–^^13^ LAIs can be part of the solution, depending on the obstacle. Four broad categories of risk factors can be conceptualized, each with several aspects to consider (Table 1). More than one factor may be present, and they may change over time. Each factor that is present requires a different strategy; for example, cognitive impairment leading to poor adherence can be remedied by additional structure and reminders (or LAIs), whereas not being able to access a pharmacy or problems storing medications in a communal setting will require quite different approaches. Community stigma can be partly addressed by public advocacy. Poor adherence is often unintentional—there are simply too many obstacles that get in the way. This is different from intentional nonadherence, where a conscious choice is being made not to follow a treatment plan; in that instance, motivational interviewing may help.

**Table 1.** Selected Risk Factors for Poor Adherence to AntipsychoticMedication

Patient-related Poor insight into the need for medications Negative attitude Prior nonadherence Substance use Cognitive impairment Treatment-related Side effects Lack of efficacy/continued symptoms Environmental- or relationship-related Lack of family/social support Problems with therapeutic alliance Practical problems with getting/storing/taking medications Societal-related Stigma attached to illness Stigma attached to medication

## What do guidelines recommend?

In a systematic review of schizophrenia clinical practice guidelines on acute and maintenance management with antipsychotics,^14^ LAIs were noted to be primarily recommended for patients who are nonadherent to other antipsychotic administration routes, and a smaller number of guidelines also suggested that LAIs should be prescribed based on patient preference. Most guidelines recommended LAIs as maintenance therapy, and some also recommended LAIs specifically for patients experiencing a first episode. The American Association of Community Psychiatrists Guidelines^15^ suggest that LAIs may offer a more convenient mode of administration or potentially address other clinical and social challenges, as well as provide more consistent plasma levels. A summary of recommendations for LAIs in the treatment of schizophrenia is provided in Table 2.

**Table 2.** Recommendations for LAI antipsychotics in the treatment of schizophrenia

Guideline	Use for a first-episode schizophrenia	Use for maintenance treatment	Use for nonadherence	Use based on patient preference
APA, 2021	Not reported	Not reported	Yes	Yes
Florida Medicaid Program, 2020	Yes	Yes	Yes	Yes
BAP, 2020	No	Yes	Yes	Yes
Oregon Health Authority, 2019	Not reported	Yes	Yes	Yes
PPA, 2019	Not reported	Not reported	Yes	Not reported
AACP, 2017	Not reported	Yes	Yes	Yes
CPA, 2017	Not reported	Not reported	Not reported	Yes
UNHCR, 2017	Not reported	Not reported	Yes	Not reported
WFSBP, 2012, 2013, 2017	Not reported	Yes	Not reported	Not reported
RANZCP, 2016	Yes	Yes	Yes	Yes
NICE, 2014	No	Yes	Yes	Yes
AFPBN, 2013	Yes	Yes	Not reported	Yes
CINP, 2013	Not reported	Not reported	Yes	Not reported
SIGN, 2013	No	Yes	Yes	Yes
Singapore Ministry of Health, 2011	No	Yes	Yes	Yes
Schizophrenia PORT, 2010	No	Yes	Not reported	Yes
Italian Guidelines, 2008	NA	NA	NA	NA
TMAP, 2008	Yes	Yes	Yes	Not reported
NJDMHS, 2005	Yes	Yes	Yes	Not reported

*AACP*, American Association of Community Psychiatrists; *AFPBN*, Association Française de Psychiatrie Biologique et Neuropsychopharmacologie; *APA*, American Psychiatric Association; *BAP*, British Association of Psychopharmacology; *CINP*, International College of Neuropsychopharmacology; *CPA*, Canadian Psychiatric Association; *LAI*, long-acting injectable; *no*, not recommended; *NA*, not applicable; *NICE*, National Institute for Health and Care Excellence; *NJDMHS*, New Jersey Division of Mental Health Services; *PPA*, Polish Psychiatric Association; *PORT*, Patient Outcomes Research Team; *RANZCP*, Royal Australian/New Zealand College of Psychiatrists; *SIGN*, Scottish Intercollegiate Guidelines Network; *TMAP*, Texas Medication Algorithm Project; *UNHCR*, United Nations High Commissioner for Refugees; *WFSBP*, World Federation of Societies of Biological Psychiatry; *yes*, recommended.

The consensus guidelines regarding the determination that a patient has treatment-resistant schizophrenia^16^ recommend that to rule out “pseudo-resistance” due to inadequate treatment adherence, patients should receive at least one trial with a LAI, given for at least 6 weeks after it has achieved steady state (generally at least 4 months from commencing treatment).

There is less literature regarding guidelines specifically for the use of LAIs in bipolar disorder or schizoaffective disorder. The French Association for Biological Psychiatry and Neuropsychopharmacology suggested that LAIs can be considered a first-line treatment for schizoaffective disorder and a second-line treatment for bipolar disorder.^17^ General and focused guidelines regarding the treatment of bipolar disorder have mentioned the use of LAIs as first line for maintenance therapy.^18^^,^^19^

## Use of long-acting injectable antipsychotics in acute inpatient settings

LAIs are generally thought of as a long-term strategy; thus, their initiation in acute inpatient settings is not always considered despite their potential therapeutic benefits. In a retrospective study that included 94,989 hospitalizations from 2010 to 2016 throughout the United States due to schizophrenia, schizoaffective disorder, or bipolar disorder, very modest rates of LAI utilization were observed.^20^ The rate of LAI monotherapy use was 1.1%, and the rate for the combination of LAI with non-LAI medication was 10.3%. The efficacy of the acute use of LAIs has been demonstrated in a meta-analysis that included 66 studies with 16,457 participants.^21^ The American Psychiatric Association Practice Guideline for the Treatment of Patients With Schizophrenia^22^ has suggested that LAIs be considered when patients are transitioning between settings, such as when patients are being discharged from an inpatient unit (eg, at inpatient discharge, on release from a correctional facility), and also earlier in the course of schizophrenia.^23^^–^^25^ In treatment planning, it should be noted that the use of a LAI is not a panacea, as observed in a Medicaid database study of 1,312 inpatients with schizophrenia where the type of antipsychotic received was not significantly associated with probability of a follow-up visit and that substance-related disorders significantly decreased it.^26^ A potential confound is severity of illness and complexity of the medication regimen; in a retrospective cohort study of 1,197 adults with schizophrenia and who initiated LAI treatment during a psychiatric inpatient stay, patients prescribed a combination of LAI and oral medication upon discharge had a higher risk of rehospitalization compared with those prescribed a LAI alone.^27^ In a small single-center prospective study of 51 patients, more than 40% of patients receiving a LAI in the hospital did not follow up in the outpatient setting despite discharge planning.^28^ The authors concluded that before initiating a LAI, “multiple factors should be considered, including outpatient adherence, access, feasibility of outpatient continuation, and transition of care plan.”

## Use of long-acting injectable antipsychotics in Community Mental Health outpatient settings

In contrast to acute hospital settings, LAIs are more often considered part of a long-term treatment strategy. Utilization rates of LAIs have been estimated to be approximately 17% among Community Mental Health Center patients, as ascertained in a study of 41,401 patients in South Carolina.^29^ Both prospective and retrospective studies have demonstrated that LAIs outperform oral antipsychotics in decreasing rates of relapse, hospitalization, and all-cause discontinuation,^30^^,^^31^ as well as exhibiting a decrease in all-cause mortality.^32^ Several obstacles to increased use of LAIs in community settings have been identified and include a lack of advocacy, difficulty with provider buy-in, limited availability of peer specialists, and a lack of infrastructure.^33^ Nonetheless, there is an opportunity in outpatient settings to introduce the idea of LAIs to potential patients using the techniques of shared decision-making,^34^ motivational interviewing,^35^ and ongoing engagement with caregivers.^36^ Site staff training in shared decision-making and roleplaying was noted to have a success rate of almost 80% for first-episode and early-phase schizophrenia to receive at least one LAI injection in a cluster-randomized clinical trial.^23^ Patients can be accepting of LAIs, provided they are presented in a positive light with sufficient information, as demonstrated in a study of communication patterns in the offer of LAIs that included 10 community mental health clinics.^37^ In the initial encounter, only 9% of the communication of psychiatrists presenting LAIs focused on positive aspects and yielded acceptance of LAIs for 11 of the 33 communications. When re-approached with better information, among almost all those who declined, subsequently stated they would be willing to try a LAI. Identified as patient barriers to LAIs include lack of awareness, sense of coerciveness, fear of injections or needles, and limited insurance coverage.^38^^–^^40^ Identified as clinician barriers include insufficient knowledge or experience, perceived lack of time, insufficient ancillary support, overestimation of adherence, lack of confidence, and stigma.^39^

The literature regarding LAIs for the management of bipolar disorder notes that LAIs are effective and well-tolerated maintenance treatments for both bipolar disorder and schizoaffective disorder, but show better efficacy in preventing mania than depression, and thus LAIs may be first-line for bipolar disorder and schizoaffective disorder patients with a manic predominant polarity.^41^^–^^43^ An expert consensus review advocates for the earlier use of LAIs in patients with bipolar disorder, ideally at the first manic episode, to aid in improving long-term outcomes.^44^ For both schizophrenia and bipolar disorder, when patients discontinue therapy, the time to relapse is longer for LAIs compared with their oral equivalents.^45^^,^^46^

## Use of long-acting injectable antipsychotics in jails

It has been observed that severely mentally ill persons who come to the attention of law enforcement now receive their “inpatient” treatment in jails and prisons, partly due to a reduction of psychiatric inpatient beds, a phenomenon that is sometimes referred to as transinstitutionalization.^47^^,^^48^ Relatively little has been published regarding the use of LAIs in jail settings. People who are jailed may be abruptly moved or bailed out prior to arrangements for ongoing mental health care and leave without medication. In a survey of Missouri county jails, only 57% of jails were able to provide LAIs.^49^ This may be an opportunity lost, as LAIs may provide a chance to provide additional time to seek ongoing care successfully. Here, treatment planning involving LAIs will require the availability of infrastructure, as well as institutional support.

## Who is the ideal candidate for long-acting injectable antipsychotics?

As part of long-term treatment planning, any individual requiring maintenance antipsychotic treatment should be informed of the availability of LAIs as part of shared decision-making. There may be specific clinical characteristics that may further support the use of this modality.

Patient preference can be a key determinant of suitability for a LAI. In a study involving 1,402 individuals with schizophrenia, 77% reported preferring LAIs over daily oral medications, citing benefits such as improved quality of life, reduced anxiety about missed doses, and fewer disruptions to daily routines.^50^ In a survey conducted in France that included 206 patients with at least 3 months of LAI antipsychotic experience, injectable antipsychotics were the preferred formulation, and 70% of patients felt better supported in their illness by virtue of regular contact with the doctor or nurse who administered their injection.^51^

Many patients find the simplicity of monthly or quarterly injections preferable to daily oral medications, particularly if they are balancing work, travel, or personal responsibilities. Others cite relief from daily reminders of illness and reduced worry about missed doses.^50^ Offering LAIs as a standard part of treatment planning—rather than reserving them only for individuals with demonstrated nonadherence—aligns with recovery-focused, patient-centered care models and ensures equitable access to evidence-based interventions.

While LAIs offer important advantages for many patients, they are not always the right choice. For example, individuals who are adherent and stabilized on clozapine—the gold standard for treatment-resistant schizophrenia—are not candidates for a switch to a LAI. However, adding a LAI to a regimen that includes clozapine may have advantages as reported in multiple observational studies.^52^^–^^56^

## Treatment planning for olanzapine pamoate

Treatment planning for the use of olanzapine pamoate is more complex than for other LAIs.^57^ When olanzapine pamoate was being developed, a small number of adverse events termed post-injection delirium/sedation syndrome (PDSS) were found in 0.07% of injections.^58^ Because there are no clear, identifiable risk factors for PDSS, a Risk Evaluation and Mitigation Strategies (REMS) was instituted, and olanzapine pamoate can only be provided at registered healthcare facilities, where patients must be monitored by appropriately qualified staff for at least 3 hours after injection.^59^ Similar guidance is in place in other countries.^57^ In addition, patients must be accompanied to their next destination upon leaving the facility. Although PDSS is not common, from a provider perspective, a clinic with 60 patients receiving an injection every 2 weeks might expect approximately one event per year.^60^ Thus, treatment planning for olanzapine pamoate will routinely require more intensive staff care and management compared with other LAIs. An alternate formulation of olanzapine LAI, administered subcutaneously and likely free of risk for PDSS, is in the late stage of clinical development.^61^

## Do formulary restrictions get in the way?

In a study of 493,006 people with schizophrenia living in the community conducted during 2002–2006, it was estimated that approximately 90% were covered by Medicare or Medicaid (with 26% having both), and approximately 7% were uninsured.^62^ Prior authorization^63^^,^^64^ and step therapy not only can delay the initiation of LAIs but also alter clinical decisions, with step therapy having the biggest impact on prescribing.^64^ However, a report of formulary restrictions in several thousand Medicare and dual Medicare/Medicaid plans in the United States, as recorded in 2019 and 2023, found that requirements for a prior authorization for LAIs (with the exception of olanzapine pamoate) were low (<12%) and declined between 2019 and 2023, and that requirements for step therapy were low as well (<4%).^65^ Medicare recipients generally (>90%) have low out-of-pocket costs.^66^ Thus, at least for the Medicare and Medicare–Medicaid population, fulfillment of a prescription for a LAI is not a major barrier for use.

## Augmentation with psychosocial interventions

Treatment planning for LAIs can benefit from the consideration of psychoeducational strategies. In a meta-analysis that included 12 studies and 874 patients, the cumulative relapse rate was 28% for those receiving family intervention compared with 49% for usual care.^67^ Another meta-analysis that included 1,534 participants across 18 studies concluded that interventions that included families were more effective in reducing symptoms than interventions directed at patients alone.^68^ Although these studies did not necessarily include LAIs in either treatment group, as a general rule, elements of psychoeducation, often in conjunction with family members or other individuals who engage in the patient’s life, are an integral part of good clinical practice.^22^ Family members can serve as strong advocates for the use of LAIs, as evidenced by the finding that caregivers generally report having fewer barriers caring for patients receiving LAIs than caring for patients not receiving such treatments.^36^

## Conclusion

In the management of schizophrenia, bipolar I disorder, and schizoaffective disorder, long-term treatment success depends not only on acute symptom control but also on maintaining clinical stability over time. Relapse prevention, sustained engagement with care, and minimizing interruptions in treatment are foundational to achieving these goals. LAIs offer an evidence-based option to support these outcomes. However, despite these advantages, LAIs remain underutilized. Treatment planning should include the routine consideration of LAIs at all stages of illness. Proactively offering LAIs as part of ongoing shared decision-making allows clinicians and patients to align pharmacologic care with individual needs and preferences—whether those preferences are shaped by practical considerations, previous treatment experiences, or lifestyle factors. Framing LAIs as a collaborative option—not as a reactive corrective measure—can reduce stigma and broaden access to a treatment modality that supports long-term stability.

## Data Availability

This article is based on previously published studies and does not report any new data. Therefore, no datasets were generated or analyzed.

## CNS SPECTRUMS

### CME Review Article

#### Understanding Long Acting Injectable Antipsychotics in the Context of Treatment Planning: Crafting a Strategy

*This CME activity is provided by HMP Education and Neuroscience Education Institute (NEI).*

### CME/CE Information

**Target Audience:** This activity has been developed for the healthcare team or individual prescriber specializing in mental health. All other healthcare team members interested in psychopharmacology are welcome for advanced study.

**Learning Objectives:** After completing this educational activity, you should be better able to:

- Become familiar with the potential benefits of LAI treatment, including convenience and improved outcomes
- Describe various patient types that are appropriate for and could benefit from LAI treatment

**Accreditation:** In support of improving patient care, this activity has been planned and implemented by HMP Education and Neuroscience Education Institute (NEI). HMP Education is jointly accredited by the Accreditation Council for Continuing Medical Education (ACCME), the Accreditation Council for Pharmacy Education (ACPE), and the American Nurses Credentialing Center (ANCC), to provide continuing education for the healthcare team.

**Activity Overview:** This activity is best supported via a computer or device with current versions of the following browsers: Mozilla Firefox, Google Chrome, or Safari. A PDF reader is required for print publications. A post-test score of 70% or higher is required to receive CME/CE credit.
**Estimated Time to Complete:** 1 hour.

Continuing Education credit will be available for three (3) years from the publication date of the associated article. Please visit https://nei.global/cnsspectrums2025 for additional information and to access the CE activity.

**NEI maintains a record of participation for six (6) years.*

#### Instructions for Optional Posttest and CME Credit

1. Read the article
2. Successfully complete the posttest at https://nei.global/CNS/LAI-01
3. Print your certificate

Questions? Email customerservice@neiglobal.com.

**Credit Designations:** The following are being offered for this activity:

- **Physician: ACCME *AMA PRA Category 1 Credits*™**
  - HMP Education designates this enduring material for a maximum of 1.00 *AMA PRA Category 1 Credit*™. Physicians should claim only the credit commensurate with the extent of their participation in the activity.
- **Nurse: ANCC contact hours**
  - This continuing nursing education activity awards 1.00 contact hour. Provider approved by the California Board of Registered Nursing, Provider #18006 for 1.00 contact hour.
- **Nurse Practitioner: ACCME *AMA PRA Category 1 Credit*™**
  - American Academy of Nurse Practitioners National Certification Program accepts *AMA PRA Category 1 Credits*™ from organizations accredited by the ACCME.
  - The content in this activity pertaining to pharmacology is worth 1.00 continuing education hour of pharmacotherapeutics.
- **Pharmacy: ACPE application-based contact hours**
  - This internet enduring, knowledge-based activity has been approved for a maximum of 1.00 contact hour (.10 CEU).
  - *The official record of credit will be in the CPE Monitor system. Following ACPE Policy, NEI and HMP Education must transmit your claim to CPE Monitor within 60 days from the date you complete this CPE activity and are unable to report your claimed credit after this 60-day period. Ensure your profile includes your DOB and NABP ID.*
- **Physician Associate/Assistant: AAPA Category 1 CME credits**
  - HMP Education has been authorized by the American Academy of PAs (AAPA) to award AAPA Category 1 CME credits for activities planned in accordance with the AAPA CME Criteria. This internet enduring activity is designated for 1.00 AAPA Category 1 credit. Approval is valid until February 11, 2028. PAs should only claim credit commensurate with the extent of their participation.
- **Psychology: APA CE credits**
  - Continuing Education (CE) credits for psychologists are provided through the co-sponsorship of the American Psychological Association (APA) Office of Continuing Education in Psychology (CEP). The APA CEP Office maintains responsibility for the content of the programs. This activity awards 1.00 CE Credit.
- **Social Work: ASWB-ACE CE credits**
  - As a Jointly Accredited Organization, HMP Education is approved to offer social work continuing education by the Association of Social Work Boards (ASWB) Approved Continuing Education (ACE) program. Organizations, not individual courses, are approved under this program. Regulatory boards are the final authority on courses accepted for continuing education credit. Social workers completing this internet enduring course receive 1.00 general continuing education credit.
- **Non-Physician Member of the Healthcare Team: Certificate of Participation**
  - HMP Education awards hours of participation (consistent with the designated number of *AMA PRA Category 1 Credit*™) to a participant who successfully completes this educational activity.

**Peer Review:** The content was peer-reviewed by a DO specializing in depression, bipolar, anxiety, and TMS — to ensure the scientific accuracy and medical relevance of information presented and its independence from commercial bias. NEI and HMP Education take responsibility for the content, quality, and scientific integrity of this CME/CE activity.

**Disclosures:** All individuals in a position to influence or control content are required to disclose any relevant financial relationships. Any relevant financial relationships were mitigated prior to the activity being planned, developed, or presented.
***Faculty Author / Presenter***

**Leslie Citrome, MD, MPH**
*Clinical Professor of Psychiatry and Behavioral Sciences, New York Medical College, Valhalla, NY* Consultant/Advisor: AbbVie/Allergan, Acadia, Adamas, AdhereTech, Alkermes, Alumis, Angelini, Astellas, Autobahn, Avanir, Axsome, Biogen, BioXcel, Bristol-Myers Squibb, Boehringer Ingelheim, Cadent Therapeutics, Cerevel, Clinilabs, COMPASS, Delpor, Draig Therapeutics, Eisai, Enteris BioPharma, HLS Therapeutics, Idorsia, INmune Bio, Impel, IntraCellular Therapies, Janssen, Karuna, Lundbeck, Luye, Lyndra, MapLight,Marvin, Medavante-ProPhase, Merck, Mitsubishi-Tanabe Pharma, Neumora, Neurocrine, Neurelis, Noema, Novartis, Noven, Otsuka, Ovid, Praxis, Recordati, Relmada, Reviva, Sage, Sumitomo/Sunovion, Supernus, Teva, The University of Arizona, Vanda, and Wells Fargo, and has provided one-off ad hoc consulting for individuals/entities conducting marketing, commercial, or scientific scoping research Speakers Bureau: AbbVie/Allergan, Acadia, Alkermes, Angelini, Axsome, BioXcel, Bristol-Myers Squibb, Eisai, Idorsia, Intra-Cellular Therapies, Janssen, Lundbeck, Neurocrine, Neopharm, Noven, Otsuka, Recordati, Sage, Sunovion, Takeda, Teva, Vanda, and CME activities organized by medical education companies such as Medscape, NACCME, NEI, Vindico, and Universities and Professional Organizations/Societies. Stockholder: owns stocks (a small number of shares of common stock) in Bristol-Myers Squibb, Eli Lilly, Johnson & Johnson, Merck, and Pfizer purchased more than 10 years ago Stock Options: Reviva Royalties/Publishing: Taylor & Francis (Editor-in-Chief, Current Medical Research and Opinion, 2022–date), Wiley (Editor-in-Chief, International Journal of Clinical Practice, through the end of 2019), UpToDate (reviewer), Springer Healthcare (book), and Elsevier (Topic Editor, Psychiatry, Clinical Therapeutics, through Spring 2025)
**Desiree Matthews, PMHNP-BC**
*CEO/Founder, Different MHP PC Charlotte, NC* Consultant/Advisor: AbbVie, Alkermes, Biogen/Sage, Boehringer Ingelheim, Bristol Myers Squibb, Indivior, Johnson and Johnson, Neurocrine Biosciences, Otsuka Pharmaceuticals, Teva Pharmaceuticals Speakers Bureau: AbbVie, Bristol Myers Squibb, Johnson and Johnson, Lundbeck, Neurocrine Biosciences, Otsuka Pharmaceuticals, Teva Pharmaceuticals The remaining Planning Committee members, Content Editors, Peer Reviewer, and NEI planners/staff have no financial relationships to disclose. NEI and HMP Education planners and staff include Meghan Grady, Caroline O’Brien, MS, Ali Holladay, Moriah Carswell, Andrea Zimmerman, EdD, CHCP, Brielle Calleo, and Jonathan Becker, DO.

**Disclosure of Off-Label Use:** This educational activity may include discussion of unlabeled and/or investigational uses of agents that are not currently labeled for such use by the FDA. Please consult the product prescribing information for full disclosure of labeled uses.

**Cultural Linguistic Competency and Implicit Bias:** A variety of resources addressing cultural and linguistic competencies and strategies for understanding and reducing implicit bias can be found in this handout—download me.

**Accessibility Statement**

For questions regarding this educational activity, or to cancel your account, please email customerservice@neiglobal.com.

**Support:** This activity is supported by an unrestricted educational grant from Alkermes, Inc., Teva Pharmaceuticals, Johnson & Johnson Innovative Medicine and Otsuka America Pharmaceutical, Inc.
